# Respiratory responses and isocapnic buffering phase in child and youth soccer players during an incremental exercise test

**DOI:** 10.3389/fphys.2024.1407759

**Published:** 2024-09-23

**Authors:** Selcen Korkmaz Eryılmaz, Selçuk Karakaş, Cumhur Boyraz, Özgür Günaştı, Abdullah Kılcı, Çiğdem Özdemir, Kerem Özgünen, Muhammed Koç, Ümit Adaş, Sadi Kurdak

**Affiliations:** ^1^ Department of Physical Education and Sports Teaching, Faculty of Sports Sciences, University of Cukurova, Adana, Türkiye; ^2^ Department of Physical Education and Sports Teaching, Faculty of Sports Sciences, University of Gedik, Istanbul, Türkiye; ^3^ Department of Coaching Education, Faculty of Sports Sciences, University of Cukurova, Adana, Türkiye; ^4^ Department of Physiology, Faculty of Medicine, University of Cukurova, Adana, Türkiye; ^5^ Department of Coaching Education, Faculty of Sports Sciences, University of Aksaray, Aksaray, Türkiye

**Keywords:** metabolic threshold, respiratory compensation point, oxygen uptake, ventilation, cardiopulmonary exercise testing

## Abstract

**Purpose:**

This study investigated the respiratory response and isocapnic buffering (IB) phase during an incremental exercise test to exhaustion in 16 child soccer players (11.9±0.9 years) and 18 youth soccer players (18.2±2.9 years).

**Methods:**

The IB phase was calculated as the difference in oxygen uptake (VO2) between the respiratory compensation point (RCP) and metabolic threshold (MT) and expressed in either absolute or relative values.

**Results:**

The maximal oxygen uptake (VO_2max_) was higher in youth players than in child players. For youth players, VO_2max_ was measured at 55.9 ± 3.6 mL min^−1^ kg^−1^ and 74.9 ± 4.8 mL min^−1^ kg^−0.75^, while for child players, VO_2max_ was 50.8 ± 4.1 mL min^−1^ kg^−1^ and 67.2 ± 6.1 mL min^−1^ kg^−0.75^ (*p* < 0.001). MT and RCP occurred at 69.8 ± 6.7% and 90.9 ± 6.9% of VO_2max_ in child players and at 73.9 ± 5.1% and 91.5 ± 4.5% of VO_2max_ in youth players, respectively. The two groups had no significant difference (p > 0.05). Absolute IB (10.6 ± 2.8 vs 9.7 ± 3.1 mL min^−1^ kg^−1^), relative IB (23.1 ± 5.7 vs 19.1 ± 6.1), and the ratio of RCP VO_2_ to MT VO_2_ (1.3 ± 0.09 vs 1.24 ± 0.09) were similar in child and youth players (*p* > 0.05). There was no difference in minute ventilation (V̇E, mL min^−1^ kg^−1^) and respiratory exchange ratio during exercise between the two groups (*p* > 0.05). During exercise, respiratory frequency, ventilatory equivalent for carbon dioxide (VE/VCO_2_) and oxygen (VE/VO_2_), VE/VCO_2_ slope, end-tidal O_2_ pressure were higher in child players than in youth players, while tidal volume (L kg^−1^), O_2_ pulse, and end-tidal CO_2_ pressure were lower (p < 0.05).

**Conclusion:**

Despite differences in aerobic capacity and ventilatory response to exercise, child players showed similar IB phase as youth players. Although child players have lower ventilation efficiency than youth players, the higher ventilation response for a given VCO_2_ may provide an advantage in regulating acid-base balance during intense exercise.

## Introduction

Maximal oxygen uptake (VO_2max_), ventilatory anaerobic threshold (metabolic threshold), and respiratory compensation point (RCP) are important physiological measurements used to determine aerobic fitness, training adaptations, and training planning in soccer players (Cunha et al., 2016). Metabolic threshold (MT) based on gas exchange measurements is considered the point at which the contribution of anaerobic metabolism to overall metabolism begins to increase during incremental exercise (Wasserman et al., 2012). When MT is exceeded during incremental exercise testing, non-oxidative CO_2_ production increases due to H^+^ buffering by blood bicarbonate (Wasserman et al., 2012). As exercise intensity increases further, the buffering system eventually becomes overwhelmed, causing a decrease in blood pH and further stimulation of hyperventilation (Binder et al., 2008; Oshima et al., 1998; Wasserman et al., 2012). This induces a second ventilatory threshold called the respiratory compensation point (RCP) (Meyer et al., 2004; Whipp et al., 1989). Physiologically, RCP marks the failure of the body’s buffering mechanism (Meyer et al., 2004). The period between MT and RCP is called the isocapnic buffering (IB) and represents the compensation phase for exercise-induced metabolic acidosis (Takano, 2000; Whipp et al., 1989). The period between the RCP and the exhaustion point of the exercise is called the hypocapnic hyperventilation (HHV) phase (Chicharro et al., 2000; Oshima et al., 1998).

The IB phase is assumed to be an indicator of cardiopulmonary function in athletes, healthy individuals, and patients with cardiac diseases (Yen et al., 2018). Although the IB phase is considered an indicator of endurance performance in healthy individuals and athletes (Oshima et al., 1998), it appears to be more associated with performance in activities that lead to a pronounced elevation in blood lactate and accompanying metabolic acidosis (Bentley et al., 2005). The range of the IB phase is related to factors such as buffering capacity, lactate kinetics, and sensitivity of the carotid bodies (Whipp et al., 1989). These factors are adaptive variables, and their response to physical training depends on exercise planning (e.g., intensity and volume) (Edge et al., 2006; Hasanli et al., 2015). Therefore, the IB phase can be a useful tool for coaches and physiologists to characterize metabolic adaptations induced by a specific training regimen (Hasanli et al., 2015). Although the IB phase decreases with age, it is longer in trained athletes independent of age (Lenti et al., 2011). While differences in respiratory response to maximal exercise have been documented between children and adults (Prado et al., 2010; Cooper et al., 1987; Weston et al., 2021), the IB phase has not received attention in children.

Ventilatory responses to exercise in the pediatric population appear to be maturity-dependent (Giardini et al., 2011; Rowland and Cunningham, 1997). Compared to teenagers, younger children have been reported to ventilate more during exercise to eliminate a given amount of CO_2_ to maintain PaCO_2_ at a lower level (Cooper et al., 1987; Gratas-Delamarche et al., 1993; Nagano et al., 1998). Smaller body size, smaller CO_2_ storage capacity, and immaturity of the respiratory center are considered responsible for age-related differences (Ohuchi et al., 1999; Cooper et al., 1987; Weston et al., 2021). Compared to adults, children show a greater increase in VE relative to VCO_2_ during exercise, reflecting lower ventilation efficiency (Takken et al., 2020; Giardini et al., 2011). Children’s respiratory centers have been suggested to be more sensitive to CO_2_ during exercise than adults (Gratas-Delamarche et al., 1993; Nagano et al., 1998). However, ventilatory regulation related to the change in acid-base balance induced by lactic acidosis has been shown to be more efficient in boys than men during strenuous exercise (Ratel et al., 2002).

Although it has been shown that aging reduces the IB phase (Lenti et al., 2011), to our knowledge, the IB phase in children has not been studied previously. Children may exhibit different IB phases compared to adults due to children’s immature glycolytic capacity (Reybrouck et al., 1985; Fellmann and Coudert, 1994). On the other hand, children have a higher ventilatory response during exercise (Cooper et al., 1987; Gratas-Delamarche et al., 1993), which may enhance the IB phase by assisting in the respiratory compensation of pH. We hypothesized that instead of VO_2max_, child soccer players might extend the IB phase by using the compensatory mechanisms mentioned above and increase their exercise tolerance compared to youth soccer players. This study aimed to examine the respiratory response and IB phase during incremental exercise in child and youth soccer players.

## Materials and methods

### Participants

Sixteen boy soccer players (11.9 ± 0.9 years, 150.6 ± 4.6 cm and 39.03 ± 5.3 kg) and eighteen youth male soccer players (18.2 ± 2.9 years, 174.5 ± 6.5 cm, 67.9 ± 7.3 kg) from the Turkish Regional Soccer League participated in the study. The child players had at least 1 year of soccer training and match experience. They had two soccer training sessions and one official match per week. The youth players had trained soccer 3–5 times weekly for at least 4 years. They also played at least one official match per week during the competitive season. Consent forms were reviewed and signed by the players and their parents in accordance with the Declaration of Helsinki. This study was approved by the Ethics Committee of the Çukurova University Faculty of Medicine (83/2018/22). Each player avoided any exercise until 24 h before the test measurement.

The age of peak linear growth (age at peak height velocity) is an indicator of somatic maturity and represents the time of maximum growth in height during adolescence. The biological maturity of child players was assessed noninvasively by calculating years from peak height velocity (PHV) using a sex-appropriate equation derived from body mass, standing height, sitting height, leg length measurements, and age (Mirwald et al., 2002). This method is a reliable (standard error of estimate ±0.592 years), nonintrusive, inexpensive, and simple way of assessing biological maturity and has the potential to be incorporated into methodologies for predicting adult height in adolescent children, free of growth-limiting diseases (Mirwald et al., 2002; Sherar et al., 2005). Maturity offset (years) was calculated by subtracting the chronological age at the time of measurement from the age in PHV, with negative values (−) interpreted as pre-PHV and positive values (+) as post-PHV. As a result of the calculations, the maturity offset of child players was found to be −1.72 ± 0.67 years.

### Incremental exercise test

The test was performed on a treadmill (HP Cosmos, Nussdorf–Traunstein, Germany), and breath-by-breath gas measurements were taken throughout the exercise using an indirect calorimetric system (PFT Cosmed, Rome, Italy) calibrated before each session. The heart rate was recorded continuously by telemetry using a heart rate monitor (Cosmed, Rome, Italy). Each player performed a standardized warm-up consisting of a 5 min run at their own pace followed by about 3 min stretching. Following the warm-up period, players performed an incremental test protocol at a constant 1% grade incline, with speed increments of 1 km h^−1^ every minute until they could no longer keep the running pace. Child players started the test at 5 km h^−1^, while youth players started at 8 km h^−1^. The athletes were instructed to run until voluntary exhaustion and given strong verbal encouragement throughout the test to elicit their best performance. Attainment of volitional exhaustion was confirmed if two of the following criteria were satisfied: 1) a plateau in VO_2_ despite increasing speed (defined as an increase of no more than 2 mL kg^−1^ min^−1^), 2) a respiratory exchange ratio (RER) above 1.10, and 3) an HR within ten beats per minute of the age-predicted maximum HR (220 – age) (Trowbridge et al., 1997). Breath-by-breath data were averaged over 10-s periods and then smoothed by eight steps, and these values were used for further analysis. The VO_2max_ was determined as the highest 10-s VO_2_ value reached during the test. Data corresponding to 1) baseline (beginning of the exercise), 2) 50% of the range from baseline to MT (50% MT), 3) MT, 4) 50% of the range from AT to RCP (50%∆ MT-RCP), 6) RCP and 6) exhaustion were used to evaluate responses to exercise. VE/VCO_2_ slope (VE *versus* VCO_2_ slope) was calculated using linear regression from the beginning of the exercise to exhaustion and from the beginning to RCP. Oxygen pulse (O_2_ pulse) was calculated as VO_2_/HR. VO_2_, VCO_2_, O_2_ pulse, VE, and tidal volume (TV) per kilogram of body mass were expressed with relative values. Additionally, VO_2_ at baseline, MT, RCP, and maximal was expressed according to an allometric scaling exponent of 0.75 for body mass (mL min^−1^ kg^−0.75^), as recommended in previous studies (Bergh et al., 1991; Cunha et al., 2008), to eliminate the effects of body mass on adults and children.

**FIGURE 1:**
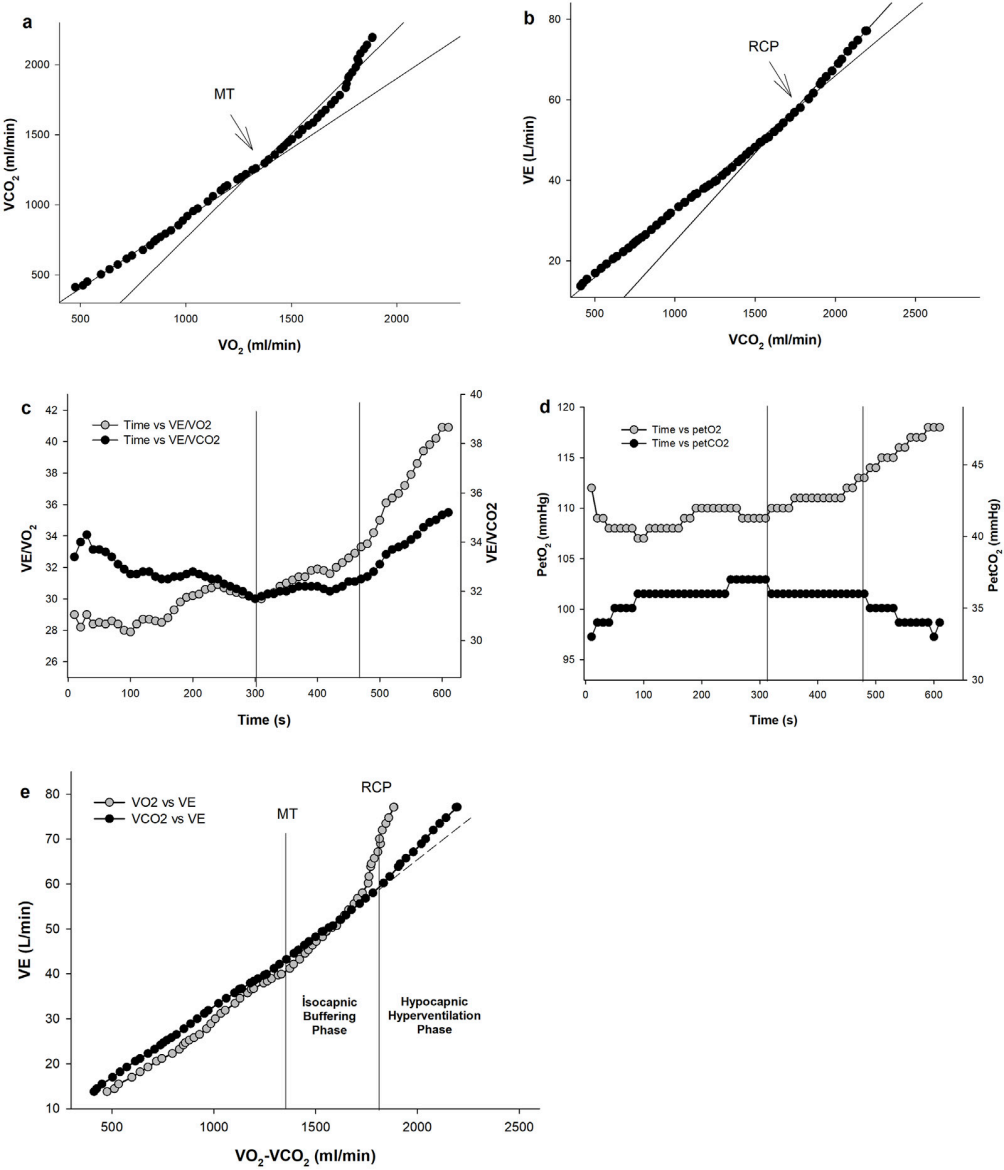
An example of gas exchange and ventilatory responses to incremental exercise in one representative child player. Determination of metabolic threshold (MT) and respiratory compensation point (RCP) using the V-slope method from VCO_2_ vs VO_2_ plot **(A)** and VE vs VCO_2_ plot **(B)**. The ventilatory equivalent for O_2_ (VE/VO_2_) and CO_2_ (VE/VCO_2_) vs time plot **(C)**, end-tidal O_2_ (PETO_2_) and CO_2_ pressure (PETCO_2_) vs time plot **(D)** were used as the secondary criteria to determine MT and RCP. Plot of ventilation (VE) vs VO_2_ and VCO_2_ **(E)** showing the isocapnic buffering and hypocapnic hyperventilation phases.

The MT and RCP were determined using the V-slope method (Beaver et al., 1986; Wasserman et al., 2012). The MT and RCP were defined as the VO_2_ value corresponding to the intersection of two linear regression lines, indicating the disproportionate increase in the VCO2 *versus* the VO_2_ and the VE *versus* the VCO_2_ relationships, respectively (Figure 1). Additionally, a visual identification technique was used to increase the identification accuracy of the MT and RCP. The MT was determined using the criterion of increases in the ventilatory equivalent for oxygen (VE/VO_2_) and end-tidal oxygen pressure (PETO_2_) without simultaneous changes in the ventilatory equivalent for carbon dioxide (VE/VCO_2_) and end-tidal carbon dioxide pressure (PETCO_2_), while the RCP corresponded to an increase in the VE/VCO_2_ and decrease in the PETCO_2_ (Wasserman et al., 2012; Binder et al., 2008). Two independent investigators performed data analysis. If there was disagreement, the opinion of a third investigator was sought.

The IB was calculated as the difference in VO_2_ between the RCP and MT and expressed as an absolute value (Oshima et al., 1998). Additionally, relative IB was calculated as previously described by Röcker et al. (1994).

$$\text{Relative IB}=\frac{\dot{V}O_{2\text{RCP}-\;}\dot{V}O_{2}\text{AT}}{\dot{V}O_{2\text{RCP}}\;}\times 100$$

where $\dot{V}O_{2}\text{RCP}$ and $\dot{V}O_{2}\text{AT}$ are the VO_2_ corresponding to RCP and AT, respectively. The HHV was calculated as the difference in VO_2_ between the VO_2max_ and the RCP and expressed in either absolute or relative values as a percentage of VO_2max_ (Oshima et al., 1998; Lenti et al., 2011). The oxygen uptake ratio of RCP to MT was calculated (RCP/MT VO_2_) (Kominami et al., 2022).

### Statistical analyses

The assumption of normality was assessed through the Shapiro-Wilk test. The two groups were compared using an independent t-test and Mann-Whitney U test for normally and non-normally distributed data, respectively. Effect sizes were calculated for independent samples using Cohen’s d method (Thalheimer and Cook, 2002). Effect sizes were interpreted as negligible (d ≥ 0.2), small (0.2 ≤ d ≤ 0.5), medium (0.5 ≤ d ≤ 0.8) or large (0.8 ≥ d). Linear regression analyses were performed using the Sigma Plot program (Sigma Plot 12.0, Systat Software Inc., Chicago, United States). IBM SPSS 21 software (IBM SPSS Statistics 21 Inc., Chicago, IL) was used for the statistical analyses. Data are reported as the means ± standard deviation (SD). Statistical significance was accepted at *p* < 0.05.

## Results

When the maturation status of child players was evaluated noninvasively, it was determined that they were in the pre-PHV period. VO_2max_, maximal VE, and exercise duration were higher in youth players compared with child players (*p* < 0.05) (Table 1). MT and RCP occurred at a similar percentage of VO_2max_ in the two groups (*p* > 0.05). The absolute and mass-adjusted relative VO_2_ and VCO_2_ values at MT, RCP, and maximal were higher in youth players than in child players (*p* < 0.05). While relative VO_2_ and VCO_2_ at baseline were higher in child players than in youth players (*p* < 0.05), the absolute values did not differ between the two groups (*p* > 0.05). VO_2_ (mL min^−1^ kg^−0.75^), expressed according to allometric scaling, was higher in youth players than in child players at MT, RCP, and maximal, while it was higher in child players than in youth players at baseline (*p* < 0.05). RER values were not different between child and youth players (*p* > 0.05). The HR values were higher at baseline in child players and at MT in youth players (*p* < 0.05) and were not different between the two groups at RCP and maximal (*p* > 0.05).

**TABLE 1:** Comparison of physiological parameters between child and youth players at baseline, anaerobic threshold, respiratory compensation point, and maximal.

Variables	Child players (n = 16)	Youth players (n = 18)	*p*	d
Baseline				
VO_2_ (mL min^−1^ kg^−1^)	14.3 ± 2.3	8.7 ± 3*	0.000	2
VO_2_ (mL min^−1^ kg^−0.75^)	0.5 ± 0.13	0.17 ± 0.05*	0.000	3.4
VO_2_ (mL min^−1^)	545.3 ± 80.7	594.4 ± 248	0.4	0.2
VCO_2_ (mL min^−1^ kg^−1^)	12.5 ± 2.4	7.8 ± 2.8*	0.000	1.7
VCO_2_ (mL min^−1^)	475.6 ± 88	533.2 ± 201	0.2	0.3
VE (L min^−1^)	16.9 ± 2.9	19.8 ± 5.8	0.07	1.2
RER	0.87 ± 0.08	0.88 ± 0.08	0.2	0.1
HR (beat min^−1^)	104.8 ± 8.1	92.7 ± 15.9*	0.01	0.9
Metabolic threshold				
VO_2_ (mL min^−1^ kg^−1^)	35.5 ± 5.2	41.3 ± 4*	0.001	1.2
VO_2_ (mL min^−1^ kg^−0.75^)	46.9 ± 7.47	55.2 ± 5.3*	0.001	1.3
VO_2_ (mL min^−1^)	1,361.1 ± 222.1	2,803.9 ± 368.2*	0.000	4.6
VCO_2_ (mL min^−1^ kg^−1^)	33.9 ± 5.3	39 ± 4.2*	0.004	1
VCO2 (mL min^−1^)	1,296.7 ± 231.6	2,642.6 ± 363.1*	0.000	4.3
VE (L min^−1^)	40.4 ± 6.8	70.5 ± 9.6	0.000	3.5
%VO_2max_	69.8 ± 6.7	73.9 ± 5.1	0.057	0.6
RER	0.95 ± 0.04	0.94 ± 0.04	0.5	0.2
HR (beat min^−1^)	152.6 ± 11.7	163.3 ± 10.6*	0.009	0.9
Running speed	8.1 ± 1.3	12.05 ± 1.2*	0.000	3.1
Respiratory compensation point				
VO_2_ (mL min^−1^ kg^−1^)	46.2 ± 5.2	51.1 ± 2.8*	0.002	1.1
VO_2_ (mL min^−1^ kg^−0.75^)	61.1 ± 7.2	68.5 ± 4.1*	0.001	1.2
VO_2_ (mL min^−1^)	1776.4 ± 229.8	3,487.5 ± 359.4*	0.000	5.6
VCO_2_ (mL min^−1^ kg^−1^)	50.5 ± 6.9	54.8 ± 4.6*	0.03	0.7
VCO_2_ (mL min^−1^)	1936 ± 317.4	3,728 ± 524.6*	0.000	4
VE (L min^−1^)	62.7 ± 10.6	101.3 ± 11.5	0.000	3.4
%VO_2max_	90.9 ± 6.9	91.5 ± 4.5	0.3	0.1
RER	1.09 ± 0.05	1.07 ± 0.07	0.3	0.3
HR (beat min^−1^)	186.4 ± 10.07	184.9 ± 5.5	0.5	0.1
Running speed	12.1 ± 1.4	15.6 ± 1.2*	0.000	2.6
Maximal				
VO_2max_ (mL min^−1^ kg^−1^)	50.8 ± 4.1	55.9 ± 3.6*	0.001	1.3
VO_2max_ (mL min^−1^ kg^−0.75^)	67.2 ± 6.1	74.9 ± 4.8*	0.000	1.4
VO_2_ (mL min^−1^)	1961.4 ± 281.8	3,811.8 ± 415.5*	0.000	5.1
VCO_2_ (mL min^−1^ kg^−1^)	59.4 ± 5	65.9 ± 5.9*	0.002	1.1
VCO_2_ (mL min^−1^)	2,286.3 ± 330.7	4,488.8 ± 715.5*	0.000	3.8
VE_max_ (L min^−1^)	80.7 ± 13.5	139.4 ± 20.8*	0.000	3.3
Exercise duration (min)	10.7 ± 1.4	12.5 ± 0.9*	0.000	1.5
RER_max_	1.19 ± 0.06	1.19 ± 0.1	0.9	0
HR_max_ (beat min^−1^)	198.8 ± 7.1	194.3 ± 6.5	0.068	0.6
Oxygen pulse (ml beat^−1^)	9.7 ± 1.4	19.5 ± 2.1*	0.000	5.4
Running speed	14.06 ± 1.1	18.5 ± 0.9*	0.000	4.4

Values are mean ± standard deviation. * Significant difference between child and youth players. P< 0.05 Max = maximal values of physiological variables, VO_2_ = oxygen uptake, VCO_2_ = carbon dioxide output, HR, heart rate; RER , respiratory exchange ratio; VE, minute ventilation.

**FIGURE 2:**
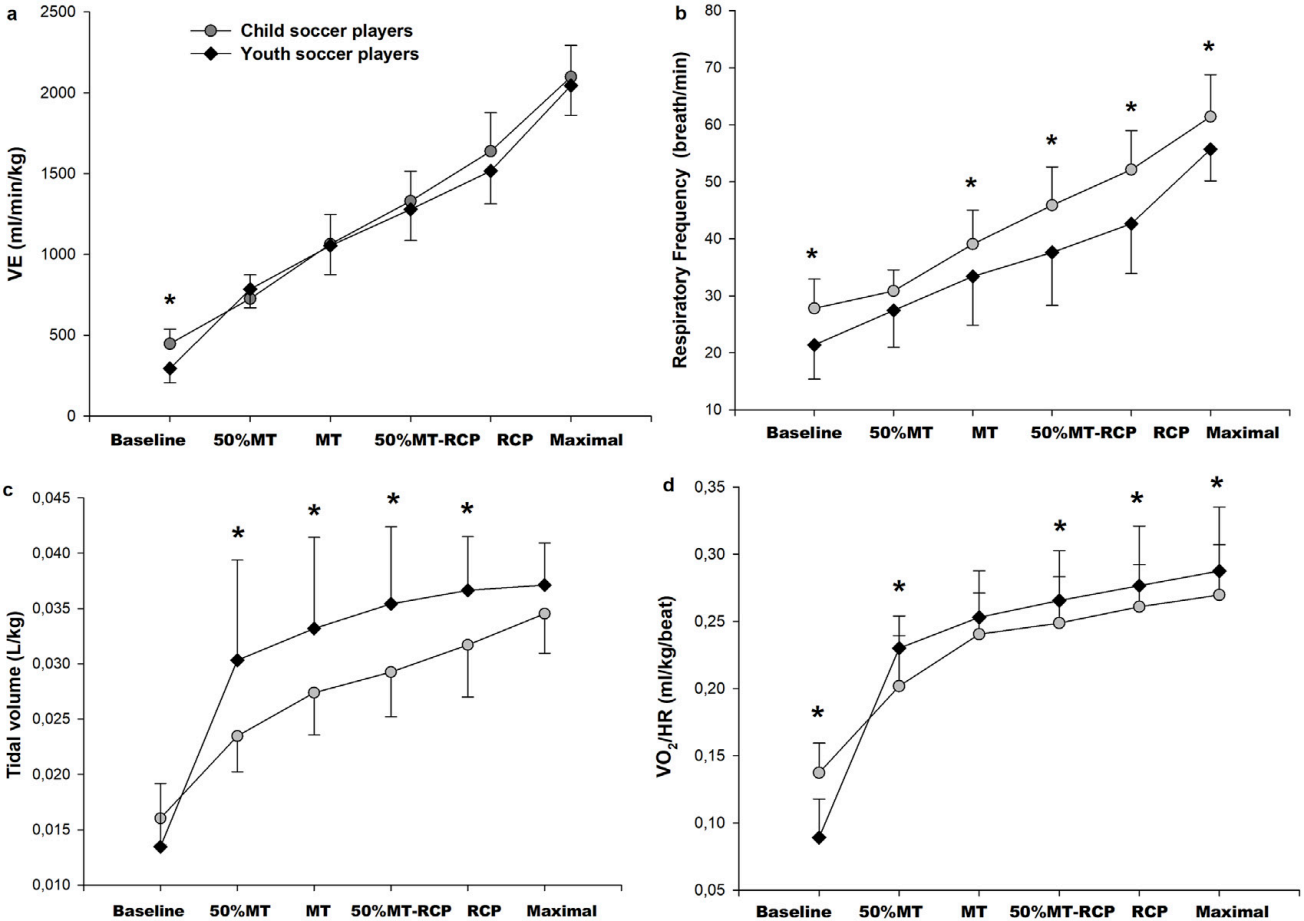
Mass-adjusted relative minute ventilation (VE) **(A)**, respiratory frequency **(B)**, relative tidal volume **(C)**, O_2_ pulse (VO_2_ HR^−1^ mL kg^−1^ beat^−1^) **(D)** responses to incremental exercise to exhaustion in child players (n = 16; gray circles) and youth players (n = 18; black square). *Significant difference between child and youth players, *p* < 0.05.

Relative VE was higher in child players at baseline (*p* < 0.05), but there was no difference between the two groups at 50%MT, MT, 50%∆MT-RCP, RCP, and maximal (*p* > 0.05) (Figure 2). Youth players had greater relative TV than child players at 50%MT, MT, 50%∆MT-RCP, and RCP (*p* < 0.05). Child players had higher respiratory frequency than youth players at baseline, MT, 50%∆MT-RCP, RCP, and maximal (*p* < 0.05). Relative O_2_ pulse was higher in child players at baseline and higher in youth players at 50%MT, 50%∆MT-RCP, RCP, and maximal (*p* < 0.05).

**FIGURE 3:**
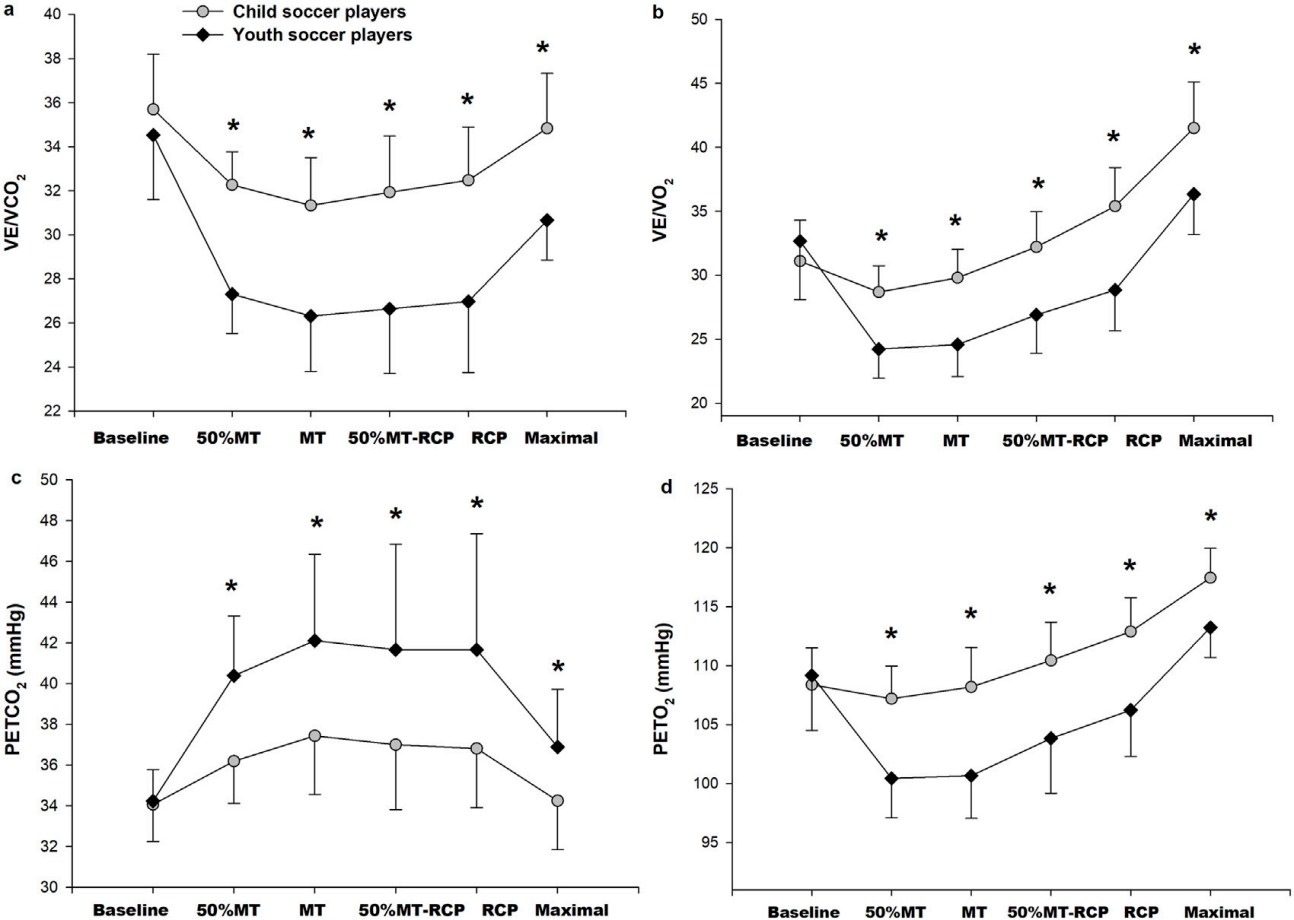
Ventilatory equivalent for CO_2_ (VE/VCO_2_) **(A)**, Ventilatory equivalent for O_2_ (VE/VO_2_) **(B)**, end-tidal CO_2_ pressure (PETCO_2_; **(C)** and end-tidal O_2_ pressure (PETO_2_) **(D)** responses to incremental exercise to exhaustion in child players (n = 16; gray circles) and youth players (n = 18; black square). *Significant difference between child and youth players, *p* < 0.05.

Child players had lower PETCO_2_ and higher PETO_2_, VE/VCO_2_, and VE/VO_2_values compared to youth players during exercise (*p* < 0.05), with no difference between the two groups at baseline (*p* > 0.05) (Figure 3). There were no significant differences between child and youth players in RCP/MT VO_2_, absolute and relative IBP, and HHP (*p* > 0.05) (Table 2). VE/VCO_2_ slope and VE/VCO_2RCP_ slope were higher in child players than in youth players (*p* < 0.05).

**TABLE 2:** Comparison of VE *versus* VCO_2_ slope, isocapnic buffering, and hypocapnic hyperventilation between child and youth players.

Variables	Child players (n = 16)	Youth players (n = 18)	*p*	d
VE/VCO_2_ slope	34.9 ± 4.4	29.5 ± 2.8*	0.000	1.4
VE/VCO_2RCP_ slope	32.02 ± 3.4	25.2 ± 3.9*	0.000	1.8
Absolute IB (mL min^−1^ kg^−1^)	10.6 ± 2.8	9.7 ± 3.1	0.3	0.3
Relative IB (%)	23.1 ± 5.7	19.1 ± 6.1	0.06	0.6
RCP/MT VO_2_	1.3 ± 0.09	1.24 ± 0.09	0.07	0.6
Absolute HHV (mL min^−1^ kg^−1^)	4.5 ± 3.3	4.7 ± 2.7	0.8	0.06
Relative HHV (%)	9 ± 6.9	8.4 ± 4.5	0.7	0.1

Values are mean ± standard deviation. *Significant difference between child and youth players, *p* < 0.05. VE/VCO_2_ slope = the VE, *versus* VCO_2_ slope from the beginning of exercise to exhaustion, VE/VCO_2RCP_, slope = the VE, *versus* VCO_2_ slope from the beginning of exercise to RCP, IB, isocapnic buffering; HHV, hypocapnic hyperventilation, RCP/MT VO_2_ = Oxygen uptake ratio of RCP, to MT., Relative IB, and HHV, were expressed as RCP, and VO_2max_ percentages, respectively.

## Discussion

The main findings of the present study are as follows: 1) child players showed an IB phase similar to youth players, 2) child players had lower aerobic capacity than youth players, and 3) child players had different ventilatory responses to incremental exercise compared to youth players. The results confirmed our hypothesis and indicated that child players exhibited similar IB phases despite having lower relative VO_2max_ compared to youth players. MT and RCP occurred at a similar VO_2max_ fraction in child and youth players, suggesting that both thresholds significantly impact the IB phase.

Respiratory frequency, VE/VCO_2_ slope, ventilatory equivalent for CO_2_ and O_2_, and PETO_2_ values of child players were higher, while TV and PETCO_2_ values were lower than youth players. These findings may be interpreted as child players’ lower ventilation efficiency during exercise than youth players (Ohuchi et al., 1999; Takken et al., 2020). Similar to the findings of previous studies (Prado et al., 2010; Gratas-Delamarche et al., 1993), the VE/VCO_2_ slope and the VE/VCO_2_ were found to be higher in child players than in youth players. Even though VE relative to body mass was not different between the two groups, lower relative VO_2_ and VCO_2_ values of child players throughout exercise indicate increased ventilatory response during exercise. Consistent with our findings, lower TV in children compared with adults during exercise is compensated by a higher respiratory frequency (Takken et al., 2020; Rowland and Cunningham, 1997; Prado et al., 2010). The lower PETCO_2_ observed in child players in this study compared to youth players during incremental exercise is consistent with previous studies (Prado et al., 2010; Ellis et al., 2017; Nagano et al., 1998; Cooper et al., 1987; Weston et al., 2021). Higher central ventilatory neural drive for children may lead to higher ventilatory rates for a given metabolic demand (Gratas-Delamarche et al., 1993).

The high VO_2max_ values of youth players indicate that they had a better aerobic capacity than child players. VO_2max_ may increase with biological maturation in regularly exercising individuals (Paterson et al., 1987; Baxter-Jones et al., 1993). Since child players have a shorter soccer training history than youth players, their lower VO_2max_ may be an expected finding. Two other important variables related to aerobic fitness, MT and RCP, occurred at similar VO_2max_ fractions in child and youth players. Consistent with our findings, studies have shown that relative RCP values in boys are similar to youth men (Cunha et al., 2008) or men (Klentrou et al., 2006), corresponding to approximately 80%–91% of VO_2max_ (or VO_2peak_) (Weston et al., 2021; Cunha et al., 2008; Klentrou et al., 2006). In children, MT varies widely, with approximately 58%–73% of VO_2max_ (Cunha et al., 2008; Paterson et al., 1987; Reybrouck et al., 1985; Mahon et al., 1998; Mahon and Vaccaro, 1989). However, active boys have a higher ventilatory anaerobic threshold than less active boys (Reybrouck et al., 1985; Weymans and Reybrouck, 1989). Although some studies have reported that MT occurs at a higher percentage of VO_2max_ in children than in adults (Klentrou et al., 2006; Weston et al., 2021), other studies similar to our study found no difference in MT between groups (Mahon et al., 1998; Cunha et al., 2016). Individuals’ training status and training history from childhood appear to play an important role in MT values (Paterson et al., 1987). Although youth players had a twice higher weekly training volume than child players due to more training sessions, the relative intensity of each soccer training was similar. The metabolic pathways used during training sessions and matches were probably equivalent in terms of physiological strain. Since relative MT and RCP were similar between child and youth players, the physiological mechanisms that identify these thresholds may differ for these age groups, and exercise intensity may be the main determinant of these two variables.

During the IB phase, both aerobic and anaerobic systems simultaneously produce energy at varying rates (Hasanli et al., 2015). If the aerobic metabolic rate is relatively high after MT, the duration of the IB phase may be longer due to the slow increase in metabolic acidosis (Yen et al., 2018). The IB phase of youth soccer players is consistent with previous research on trained men (Lenti et al., 2011; Chicharro et al., 2000; Eryılmaz et al., 2018; Korkmaz Eryılmaz et al., 2018). We found that even though youth players had higher VO_2max_, the absolute and relative IB range was similar to that of child players. In addition to that, the ratio of RCP VO_2_ to MT VO_2_ can be used to compare buffering capacity between groups of different ages (Kominami et al., 2022), and we did not find any significant difference in this ratio between youth and child players in our study. The present and previously published data (Hasanli et al., 2015; Korkmaz Eryılmaz et al., 2018; Röcker et al., 1994) indicate that the VO_2max_ value may not be enough to interpret IB and buffering capacity.

Physical training may directly affect the IB phase by changing MT and RC (Kominami et al., 2022; Lenti et al., 2011; Chicharro et al., 2000; Eryılmaz et al., 2018). Although there was no statistically significant difference in our study, child players tend to have a larger IB phase due to their lower relative MT than youth players. High-intensity training may enhance the IB phase by shifting RCP with minimal or no change in MT (Eryılmaz et al., 2018; Oshima et al., 1998). Several studies have shown higher lactate accumulation during the IB phase in anaerobic-trained athletes than in endurance-trained athletes (Hasanli et al., 2015; Röcker et al., 1994). Although we did not directly measure blood lactate, the maximal RER may reflect the lactate concentration (Stringier et al., 1995). Unlike adults, children have a muscle metabolic profile better equipped for the aerobic energy pathway rather than the glycolytic energy pathway (Fellmann and Coudert, 1994). Therefore, maximal RER values of children at the end of exercise can be expected to be lower than adults. RER values at the same relative exercise intensities did not differ significantly between child and youth players, and our data is consistent with other studies (Ohuchi et al., 1999; Giardini et al., 2011). Even though the training history of the child players was lower than that of the youth players, both groups were training specifically for soccer. It is known that regular physical training can increase the glycolytic capacity of skeletal muscles in boys (Eriksson, 1972; Paterson et al., 1987). Since youth soccer players have a more extended training history than child players, similar RER values indicate that training modalities are important for determining metabolic profiles.

Ratel et al. (2002) demonstrated a smaller decrease in blood pH relative to an increase in lactate in boys compared with men during repeated sprint exercise. This relatively small increase in blood [H^+^] in boys was explained by higher ventilatory regulation related to the change in acid-base balance compared to men during repeated sprints (Ratel et al., 2002). Children may be better able to tolerate lactic acidosis with a higher ventilatory response for a given CO_2_ (Ratel et al., 2002). This may explain the mechanism of similar IB phases between child players with lower aerobic capacity and youth players. Future studies are necessary to explain this physiological phenomenon in child and youth athletes with matched aerobic capacities or specific training programs.

## Conclusion

Child players had lower aerobic capacity and hyperventilatory response to exercise than youth players. MT and RCP occurred at similar VO_2max_ fractions in child and youth players, and both of these groups showed similar IB phases. Respiratory frequency, ventilatory equivalent for CO_2_ and O_2_, and PETO_2_ values of child players were higher than youth players. Hyperventilation reduced PETCO_2_ values, which may correspond to a reduction of PaCO_2_. Therefore, a higher ventilatory response during exercise may help children buffer pH. Longitudinal studies are needed to confirm the responsiveness of the IB phase to physical training in children.

## Data Availability

The raw data supporting the conclusions of this article will be made available by the authors, without undue reservation.
